# Acute Genitourinary Pain and Swelling in a Child‐Look Out for the Rashes!

**DOI:** 10.1002/pdi3.70012

**Published:** 2025-06-21

**Authors:** Frankel Lin, Jasmine Huang, Sudipta Roy Chowdhury

**Affiliations:** ^1^ Yong Loo Lin School of Medicine National University of Singapore Singapore Singapore; ^2^ Department of Pediatrics KK Women's and Children's Hospital Singapore Singapore; ^3^ Duke‐National University of Singapore Medical School Singapore Singapore; ^4^ Lee Kong Chian School of Medicine Nanyang Technological University Singapore Singapore

## 1 Background

Henoch‐Schonlein purpura (HSP), also known as IgA vasculitis, is an acute immune complex mediated small‐vessel vasculitis that develops most commonly in children less than 10 years of age [1]. It is usually characterized clinically by a triad of symptoms, including palpable purpura without thrombocytopenia, abdominal pain, and arthritis or arthralgia. Other clinical manifestations of HSP can vary from asymptomatic microscopic hematuria or proteinuria to more concerning symptoms of acute kidney injury. Atypical presentations may involve the urogenital system, mainly testicular and scrotal manifestations. However, penile involvement remains a rare complication and is not commonly described [2, 3]. HSP is usually a self‐limiting disease but would require urgent treatment with corticosteroids when such complications arise. We report a case of pediatric HSP who presented with the concerning symptoms of acute urinary retention secondary to penile swelling.

## 2 Case Presentation

Our patient is a 4‐year‐old boy who presented to Children's Emergency (CE) with acute retention of urine and dysuria over 24 h. On inspection, he was irritable and in discomfort. He had tenderness over his suprapubic region and his genitalia.

**FIGURE 1:**
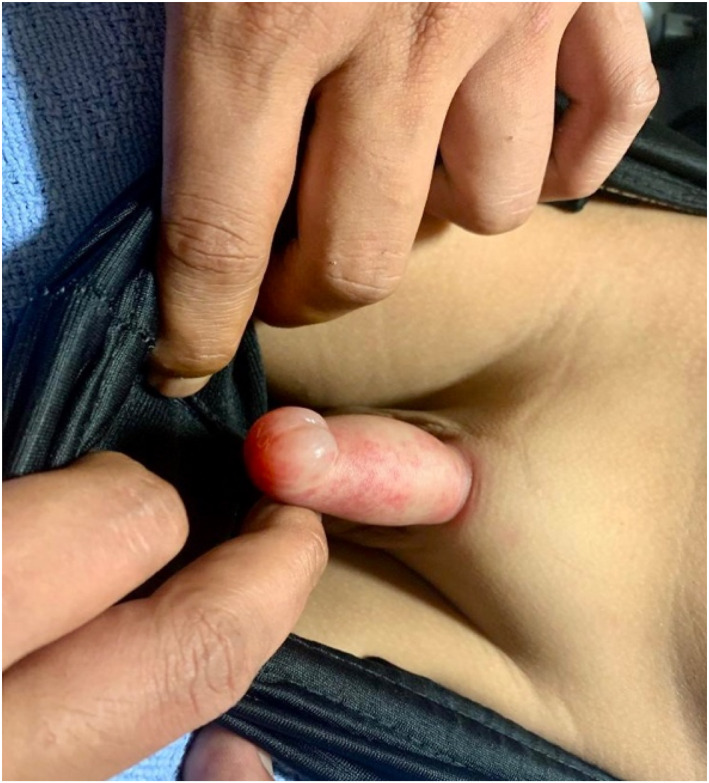
Penile purpura and ecchymosis with edema over the penile shaft.

The patient’s entire penile shaft was edematous and was tender to touch. The urethral opening was barely visualized due to surrounding edema and appeared obstructed. Outer prepuce was swollen with a large erythematous purpura over the right side of the penis and resolving purpura over the distal left side of the penis (Figure 1). The penile swelling had rapidly progressed over 48 h prior to the CE attendance. There were no abnormal urethral discharge or ulcers over his genitalia. Rest of his systemic examination was normal.

Initial investigations showed normal blood test results for full blood count and kidney function tests. Infective markers were raised (C‐reactive protein [CRP] = 16.2 mg/dL and normal range 0.2–9.1 mg/L). Urinalysis was normal with absence of blood, leucocytes, protein, and epithelial cells.

## 3 Differentials Considered

In view of his presentation of acute retention of urine, initial concerns of urinary tract infection was considered. However, he did not have urinary tract symptoms of dysuria or hematuria and his urinalysis was normal. Neurological and spinal examination was normal, and thus spinal cord involvement causing acute retention of urine was ruled out as well. Due to his prepuce swelling, balanitis was considered. Infective balanitis was the top differential as it is the most common condition. Other concerns were that of sexually transmitted infections (STIs), such as genital herpes, but this was less likely due to lack of penile discharge and absence of blisters. Furthermore, a thorough evaluation of the psychosocial aspects of this patient did not point to a concern of sexual abuse. Atypical drug allergy was considered, but there was no preceding history of new medications introduced to the child and the absence of other mucosal site involvement made this a less likely cause. He also did not have any history of contact dermatitis, which was another differential to consider. In view of the vasculitic rash, possibility of systemic vasculitis, inflammatory bowel disease (IBD), systemic juvenile idiopathic arthritis, macrophage activation syndrome, and malignancy were considered. Extraintestinal manifestations of IBD is well‐known to cause leukocytoclastic rash. However, our patient did not have any systemic symptoms or signs to suggest underlying IBD or malignancy to consider these differentials [4]. He did not have any anemia, weight loss, or chronic abdominal pain, which is usually associated with IBD.

Paraphimosis and balanoposthitis were also ruled out after a detailed genital examination, which showed diffuse penile swelling rather than primarily penile tip swelling found commonly in these conditions [5]. Other causes of acute urinary retention were ruled out, such as hypokalemia causing generalized muscle weakness, which was ruled out initially due to normal potassium levels (potassium levels = 3.7 mmol/L) and an otherwise normal neurological examination, which showed no evidence of muscle weakness. Another important cause that needs consideration for acute urinary retention is underlying renal calculi, but there were no significant risk factors (use of medications, e.g., topiramate; dehydration, metabolic conditions, and hypercalcemia) in this child. A normal renal ultrasound showed no evidence of nephrocalcinosis or abnormal echogenicity to suggest any underlying renal calculi to hint this as a possibility.

**FIGURE 2:**
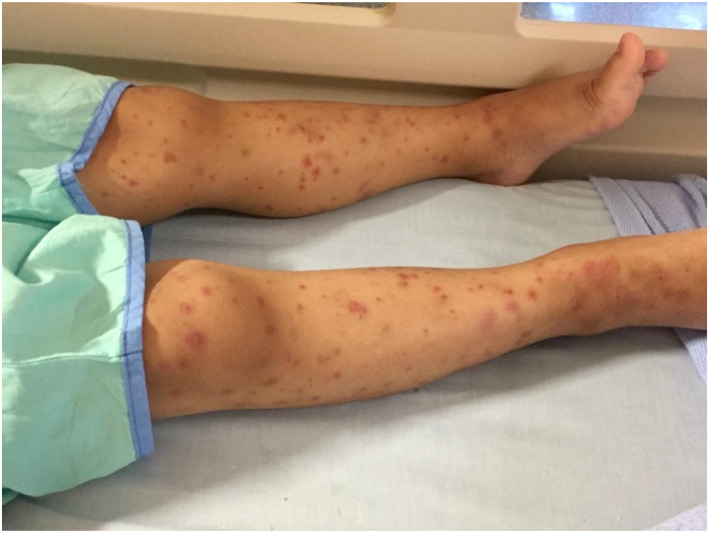
Multiple discrete maculopapular rash with palpable purpura and subcutaneous swelling of the ankles.

A more detailed history taking and physical examination revealed interesting details, which eventually clinched the diagnosis. On proper exposure of his lower limbs, patient had multiple discrete maculopapular rash with palpable purpura and subcutaneous swelling of the ankles (Figure 2). On further probing, it was revealed that he was recently diagnosed with HSP 1‐week ago. Due to this additional information, inflammatory cause of balanitis was considered instead and he was promptly admitted for a rheumatological consult and for treatment and relief of his genitourinary swelling.

The patient's acute urinary retention was a consequence of his highly edematous penile shaft causing mechanical outlet obstruction at the urethral meatus. An indwelling catheter (IDC) was inserted to bypass the obstruction and relieve the urinary retention. Further blood tests showed normal levels for serum IgA levels and erythrocyte sedimentation rate.

## 4 Progress and Outcome

Following the IDC insertion, a large amount of urine (650 mLs) was aspirated which spontaneously resolved the patient's urinary retention and abdominal pain.

After a timely rheumatological consult, he was administered intravenous pulsed methylprednisolone with tapering doses of oral steroids for treatment of HSP and its complications. Within 24 h of initiating treatment, penile swelling was markedly reduced with fewer associated purpuric rashes over the lower limbs.

He was successfully weaned off the urinary catheter within 72 h of admission as his genitourinary swelling and pain resolved.

## 5 Discussion

Cases of HSP with reported penile involvement or HSP presenting with penile rash have all been exceedingly rare. There are only few case reports of penile swelling with acute urinary retention associated with HSP [6]. In this patient, the acute management was to relieve the urine retention through a urethral catheter so as to prevent further complications such as hydronephrosis and acute kidney injury. Farkas et al. posits that suprapubic catheterization is an effective alternative when urethral catheterization was deemed too high risk [7]. Though penile involvement has been reported in several literature, it is still an extremely rare complication that deserves more attention [8]. The penile findings associated with HSP can range from edema, erythema, ecchymosis, to purpuric rash of the penile shaft or prepuce [9, 10, 11, 12]. Occasionally, these symptoms precede the findings of the distinctive purpuric rash, which makes the diagnosis even more challenging [9]. In this case, the patient presented with widespread cutaneous signs and joint arthritis, which highly suggested the impression of HSP 1 week before signs of penile involvement emerged. There have also been accounts of penile skin involvement being the first presentation of HSP, making it an even greater diagnostic challenge [13]. In such events, other differentials should be considered and excluded first.

Ferrara et al. reports that penile involvement does not confer a poorer prognosis and should be treated conservatively [14]. However, there are also other researchers, such as Pennesi et al., who report improvement with glucocorticoid treatment [15]. With regards to HSP as a whole, a systematic review by Weiss et al. provides compelling evidence that early corticosteroids reduces the need for surgical intervention and disease recurrence [16]. Our patient also had raised CRP, which is associated with higher risk of gastrointestinal bleeding in the setting of severe HSP [17, 18]. However, our patient did not have any preceding chronic abdominal pain or per‐rectal bleeding to suggest any gastrointestinal involvement. The raised CRP could be attributed to his upper respiratory tract infection that he acquired 5 days prior to the presentation of his vasculitic rash.

Due to the extensive penile swelling, our patient was treated with pulsed steroids and weaned down to a short course of oral glucocorticoids. He responded well to this treatment demonstrating the benefits of prompt treatment. It is thus critical to consider HSP as one of the differentials when there is penile swelling or balanitis‐like presentation in a child, especially in the presence of palpable purpura as timely treatment with steroids, which would then reduce further invasive treatment options and reduce the chance of further damage to the genitalia. This case report serves to highlight the rarer manifestations of HSP, beyond the classical symptoms, especially focusing on atypical edemas. Through highlighting such unusual presentations, we aim to educate and equip healthcare professionals with the knowledge to recognize and manage these rare instances effectively. Other notable reports have highlighted scalp edema as an initial manifestation of HSP [19, 20]. This emphasizes the need for clinicians to consider HSP in the differential diagnosis when presented with unusual edematous manifestations, as it underscores that HSP can lead to edemas not only in the extremities but also in unexpected areas such as the scalp and the genitalia.

It is crucial to discuss these atypical presentations to improve diagnosis and management of HSP in clinical practice. These atypical cases enhance our understanding of the various clinical presentations of HSP and underline the importance of considering atypical edemas during the diagnostic process, thereby enriching the literature on atypical manifestations of HSP.

## Author Contributions

F.L and J.H: data curation, writing of original draft preparation, and investigation. S.R.C: conceptualization, investigation, supervision, reviewing, and editing.

## Ethics Statement

Ethics approval was not required by their local institution as this is a single case report.

## Consent

Written and verbal parental consent was taken for sharing of patient’s data and for publication.

## Conflicts of Interest

The authors declare no conflicts of interest.

## Data Availability

The datasets used or analyzed during the current study are available from the corresponding author on reasonable request.
